# Chromosome‐specific KASP markers for detecting *Amblyopyrum muticum* segments in wheat introgression lines

**DOI:** 10.1002/tpg2.20193

**Published:** 2022-01-31

**Authors:** Surbhi Grewal, Benedict Coombes, Ryan Joynson, Anthony Hall, John Fellers, Cai‐yun Yang, Duncan Scholefield, Stephen Ashling, Peter Isaac, Ian P. King, Julie King

**Affiliations:** ^1^ Nottingham BBSRC Wheat Research Centre, School of Biosciences Univ. of Nottingham Loughborough UK; ^2^ Earlham Institute Norwich Research Park Norwich UK; ^3^ Current address: Limagrain Europe Clermont‐Ferrand France; ^4^ USDA‐ARS Hard Winter Wheat Genetics Research Unit Manhattan KS USA; ^5^ iDna Genetics Ltd. Norwich Research Park Norwich UK

## Abstract

Many wild‐relative species are being used in prebreeding programs to increase the genetic diversity of wheat (*Triticum aestivum* L.). Genotyping tools such as single nucleotide polymorphism (SNP)‐based arrays and molecular markers have been widely used to characterize wheat–wild relative introgression lines. However, due to the polyploid nature of the recipient wheat genome, it is difficult to develop SNP‐based Kompetitive allele‐specific polymerase chain reaction (KASP) markers that are codominant to track the introgressions from the wild species. Previous attempts to develop KASP markers have involved both exome‐ and polymerase chain reaction (PCR)‐amplicon‐based sequencing of the wild species. But chromosome‐specific KASP assays have been hindered by homoeologous SNPs within the wheat genome. This study involved whole genome sequencing of the diploid wheat wild relative *Amblyopyrum muticum* (Boiss.) Eig and development of a *de novo* SNP discovery pipeline that generated ∼38,000 SNPs in unique wheat genome sequences. New assays were designed to increase the density of *Am. muticum* polymorphic KASP markers. With a goal of one marker per 60 Mbp, 335 new KASP assays were validated as diagnostic for *Am. muticum* in a wheat background. Together with assays validated in previous studies, 498 well distributed chromosome‐specific markers were used to recharacterize previously genotyped wheat–*Am. muticum* doubled haploid (DH) introgression lines. The chromosome‐specific nature of the KASP markers allowed clarification of which wheat chromosomes were involved with recombination events or substituted with *Am. muticum* chromosomes and the higher density of markers allowed detection of new small introgressions in these DH lines.

## Core Ideas

- This study involved whole genome sequencing of wheat wild relative *Amblyopyrum muticum* for SNP discovery with wheat.
- We introduce a novel methodology to generate chromosome‐specific SNPs between wheat and its wild relatives.
- Characterization of wheat–*Amblyopyrum muticum* doubled haploid introgression lines with a high‐density KASP marker were able to detect introgressions.

**Abbreviations**
DH: doubled haploid
DNA: deoxyribonucleic acid
KASP: Kompetitive allele‐specific polymerase chain reaction
mcGISH: multi‐color genomic in situ hybridization
PCR: polymerase chain reaction
SNP: single nucleotide polymorphism
BLAST: basic local alignment search tool.

## 1 INTRODUCTION

Bread wheat (*Triticum aestivum* L., 2n = 6x = 42, AABBDD) is one of the most widely grown and consumed crops worldwide. After two spontaneous interspecific hybridization events (Dvořák et al., 1993; Marcussen et al., 2014; Pont et al., 2019), resulting in its allohexaploid genome, domestication and intensive breeding practices have reduced the genetic diversity available within and between modern bread wheat cultivars. The wild relatives of wheat, however, have a vast resource of untapped genetic variation that could be used to enrich and diversify the wheat genome. Recent studies have demonstrated the dramatic improvement in wheat–wild relative introgressions achieved through homoeologous recombination and genomics‐based marker technologies (Qi et al., 2007; Tiwari et al., 2014; King et al., 2017; Grewal et al., 2018b; Cseh et al., 2019b; Xu et al., 2020).

Detection and characterization of wild relative chromatin in a wheat background is an important requirement in wheat breeding programs. Molecular markers provide a high‐throughput and cost‐effective way of achieving this and simple sequence repeats have been a popular marker system for the detection of wild relative introgressions because of their multi‐allelic and co‐dominant nature (Wu et al., 2006; Zhao et al., 2013; Fricano et al., 2014; Niu et al., 2018). However, with recent advances in Next Generation Sequencing technologies and low‐cost genome sequencing, single nucleotide polymorphism (SNP) markers are now the front‐runner in the race to developing high‐throughput genotyping platforms for marker‐assisted selection in crop breeding (Varshney et al., 2009; Rasheed et al., 2017). Exome‐based sequencing of wheat varieties has resulted in a huge resource of SNPs (Winfield et al., 2012; Allen et al., 2013), which has been exploited to develop high‐density SNP wheat genotyping arrays (Wang et al., 2014; Winfield et al., 2016; Allen et al., 2017). Wild relative introgressions have been detected in a wheat background using such wheat‐based SNP arrays (Zhang et al., 2017; Zhou et al., 2018).

Genotyping of introgression lines is more efficient when using SNPs derived from wild‐relative genome sequences. The Axiom^®^ Wheat‐Relative Genotyping SNP Array was developed with genome‐specific SNPs from exome sequencing of various wheat wild species (Winfield et al., 2016; King et al., 2017). The array was subsequently used to detect introgressions from various wild species in a wheat background (Grewal et al., 2018a; Grewal et al., 2018b; King et al., 2018; Cseh et al., 2019a; Devi et al., 2019; Baker et al., 2020). Even though these genotyping arrays can be ultra‐high‐throughput and efficient, these SNPs cannot distinguish between homozygous and heterozygous individuals which limits their widespread use in crop breeding. Recently, whole genome and transcriptome sequencing have been used to develop genome‐specific SNPs for *Lophopyrum elongatum* (Host) Á. Löve and tools such as high‐resolution melting markers and the Sequenom MassARRAY SNP genotyping platform, utilizing these SNPs, were deployed for detecting *L. elongatum* introgressions in a wheat background (Lou et al., 2017; Xu et al., 2020).

**Core Ideas**

- This study involved whole genome sequencing of wheat wild relative *Amblyopyrum muticum* for SNP discovery with wheat.
- We introduce a novel methodology to generate chromosome‐specific SNPs between wheat and its wild relatives.
- Characterization of wheat–*Amblyopyrum muticum* doubled haploid introgression lines with a high‐density KASP marker were able to detect introgressions.

The Kompetitive allele‐specific polymerase chain reaction (KASP) platform has been demonstrated to be a flexible, efficient, and cost‐effective system for genotyping of introgression lines using wild relative genome‐specific SNPs (Bansal et al., 2020; Grewal et al., 2020b; Han et al., 2020). However, hexaploid wheat's polyploid genome makes it complicated to develop co‐dominant interspecific SNPs. The first obstacle is the distinction between interspecific and an excess of homoeologous/paralogous SNPs found within the wheat genome. The second hurdle to overcome is the scoring of interspecific SNPs in segregating populations where the SNP has three homoeologous copies in the wheat genome. In such cases, it is difficult to differentiate between a heterozygous and a homozygous introgression line in a self‐fertilized backcross population (Allen et al., 2011). Recently, Grewal et al. (2020a) addressed this problem by attempting to exploit interspecific SNPs with KASP assays that only had one copy of the template in the wheat genome. They reported wild relative genome‐specific SNPs for ten species from the *Amblyopyrum*, *Aegilops*, *Thinopyrum*, *Triticum*, and *Secale* genera using polymerase chain reaction (PCR)‐amplicon based sequencing of which 620 were validated as chromosome‐specific KASP markers in the wheat genome.

In this work, a more efficient bioinformatics‐based approach was used to develop chromosome‐specific KASP markers between *Amblyopyrum muticum* (Boiss.) Eig. (2n = 2x = 14, TT) and bread wheat. Whole genome sequence of *Am. muticum* was generated using next generation sequencing and aligned to the bread wheat cultivar Chinese Spring RefSeqv1.0 International Wheat Genome Sequencing Consortium (IWGSC) et al.,(2018) high‐quality reference genome sequence. Unlike previous work (Grewal et al., 2020a), a *de novo* approach was used for SNP discovery and a pipeline was established that filtered for those SNPs that were suitable for KASP assay design as well as those that were located in unique wheat genome sequences. A small subset of the SNPs was converted to KASP markers and validated by genotyping a previously reported panel of doubled haploid (DH) wheat–*Am. muticum* introgression lines (King et al., 2019), increasing the density of *Am. muticum* diagnostic KASP markers across the wheat genome. The methodology reported here and the resulting KASP markers can be applied to other wheat‐wild relative introgression programs and thus represents a valuable resource for the wheat research community.

## 2 MATERIALS AND METHODS

### 2.1 Plant material

Four hexaploid wheat cultivars (Chinese Spring, Paragon, Pavon76 and Highbury), three accessions of *Am. muticum* (2130004, 2130008 and 2130012; all obtained from the Germplasm Resource Unit at the John Innes Centre), three wheat–*Am. muticum* F_1_ lines (one with each accession of *Am. muticum*), and 67 doubled haploid wheat–*Am. muticum* introgression lines (King et al., 2019) were grown for leaf tissue collection and nucleic acid extraction.

All plants were grown in a glasshouse in 2L pots containing John Innes No. 2 soil and maintained at 18—25 °C under 16 h light and 8 h dark conditions. Leaf tissues (1.5‐inch leaf segments cut into pieces) were harvested from 3‐wk‐old plants, immediately frozen on liquid nitrogen, and stored at −80 °C until nucleic acid extraction.

### 2.2 Nucleic acid extraction

Leaf tissue samples were freeze‐dried in a deep‐well plate and ground in the TissueLyser II (QIAGEN) for 4–6 min at a frequency of 25 Hz. Genomic deoxyribonucleic acid (DNA) for sequencing and genotyping was extracted according to the Somers and Chao protocol (verified 10 September, 2021, original reference in Pallotta et al., 2003) from Step 2 onwards. For wild relatives with multiple accessions, the genomic DNA was pooled into one sample.

Genomic DNA extraction for generation of probes for genomic in situ hybridization analysis was carried out using the above protocol with an additional step of purification with phenol/chloroform at the end.

### 2.3 Chromosome‐specific SNP discovery

Deoxyribonucleic acid was isolated from *Am. muticum* accession 2130012, as described above, and a polymerase chain reaction (PCR)‐free library was prepared and sequenced on an Illumina HiSeq 2500 on rapid run mode to produce 101.86 Gb (∼16.50x coverage of *Am. muticum* assuming kew c‐value genome size of 6.174 Gbp) of 250 bp paired‐end reads. SNP discovery followed a bioinformatics pipeline (Supplemental Figure S1) that produced a set of SNPs suitable for KASP assay design. In detail, the reads were mapped to the wheat cultivar Chinese Spring reference genome assembly RefSeq v1.0 International Wheat Genome Sequencing Consortium (IWGSC) et al.,(2018) using BWA MEM version 0.7.13 (Li, 2013) with the ‐M flag. Alignments were filtered using SAMtools v1.4 (Li et al., 2009) to remove unmapped reads, supplementary alignments, improperly paired reads, and non‐uniquely mapping reads (q < 10) and PCR duplicates were removed using Picard's MarkDuplicates (DePristo et al., 2011). Variant calling was performed using BCFtools (Li, 2011) using the multiallelic model (‐m). Variant filtering was performed using GATK VariantFiltration (DePristo et al., 2011). SNPs passed this filtering if their depth > = 5, allele frequency (AF) > = 0.8, quality score > = 30, and they weren't in clusters of 3 or more SNPs within 10 bp. INDELs were removed and SNPs unsuitable for KASP assays were removed if any other SNP (including heterozygous SNPs or those that didn't pass the filtering) was present within 50 bp up or downstream. Heterozygous SNPs and SNPs that failed filtering were then removed. To prevent amplification of off‐target regions in the genome, the SNP site along with 50 bp up and downstream was aligned to RefSeq v1.0 using basic local alignment search tool BLASTn from the blast+ command line tool (Camacho et al., 2009) with default parameters. Queries were discarded if they produced any hits to the wheat genome except the single expected self‐hit. A detailed list of SNPs discovered is provided in Supplemental Table S1.

### 2.4 KASP assay design and genotyping

To design a KASP™ assay, the flanking sequence of a SNP was fed through the PolyMarker application (Ramirez‐Gonzalez et al., 2015) which aligned the query sequence to wheat cultivar Chinese Spring RefSeq v1.0 and provided two allele‐specific primers and one common primer for each assay. A value of 1 in the ‘*total_contigs’* column of the output *Primers* file validated the query SNP to be in a unique sequence region in the wheat genome RefSeq v1.0 assembly (Supplemental Table S2).

For genotyping purposes, three sets of KASP markers were used. Set 1 consisted of 150 chromosome‐specific KASP markers previously reported to be polymorphic between wheat and *Am. muticum* (codes between WRC0001‐1000; Grewal et al., 2020a). Set 2 consisted of 224 KASP assays designed to be tested on doubled haploid wheat–*Triticum urartu* Thumanian ex Gandilyan introgression lines (codes between WRC1080‐1308 and WRC1317‐1393; Grewal et al., 2021). This is a subset of the 304 KASP markers developed in this study after 47 failed to amplify a PCR product and 33 were polymorphic between the parental wheat cultivars. Set 3 consisted of the new KASP assays designed in this study (codes between WRC1309‐1316, WRC1394‐1713, WRC1723‐1872, WRC1894‐1913, WRC1954‐2113 and WRC2130‐2169; Supplemental Table S2).

The genotyping procedure was as described by Grewal et al. (2020b). Briefly, the genotyping reactions were set up using the automated PIPETMAX^®^ 268 (Gilson) and performed in a ProFlex PCR system (Applied Biosystems by Life Technology) in a final volume of 5 μl with 1 ng genomic DNA, 2.5 μl KASP reaction mix, 0.068 μl primer mix, and 2.43 μl nuclease‐free water. Polymerase chain reaction (PCR) conditions were set as 15 min at 94 °C; 10 touchdown cycles of 10 s at 94 °C, 1 min at 65–57 °C (dropping 0.8 °C per cycle); and 35 cycles of 15 s at 94 °C, 1 min at 57 °C. Fluorescence detection of the reactions was performed using a QuantStudio 5 (Applied Biosystems) and the data analyzed using the QuantStudio™ Design and Analysis Software V1.5.0 (Applied Biosystems).

### 2.5 Multi‐color genomic in situ hybridization

Preparation of the root‐tip metaphase chromosome spreads, the protocol for multi‐color genomic in situ hybridization (mcGISH) and the image capture was as described in Grewal et al. (2020b). Briefly, genomic DNA from *T. urartu* (to detect the A‐genome), *Aegilops speltoides* Tausch (to detect the B‐genome), *Aegilops tauschii* Coss. (to detect the D‐genome), and *Am. muticum* were isolated as described above. The genomic DNA of (a) *T. urartu* was labeled by nick translation with ChromaTide™ Alexa Fluor™ 488‐5‐dUTP (Invitrogen; C11397; colored green), (b) *Ae. speltoides* was labeled by nick translation with DEAC‐dUTP (Jena Bioscience; NU‐803‐DEAC; colored blueish purple), (c) *Ae. Tauschii* was labeled with ChromaTide™ Alexa Fluor™ 594‐5‐dUTP (Invitrogen; C11400; colored yellow), and d) *Am. muticum* was labeled by nick translation with ChromaTide™ Alexa Fluor™ 546‐14‐dUTP (Invitrogen; C11401; colored red). Slides were probed using 150 ng of *T. urartu*, 150 ng of *Ae. speltoides*, 300 ng of *Ae. tauschii*, and 50 ng of *Am. Muticum*‐labeled genomic DNAs, in the ratio 3:3:6:1 (green/blue/yellow/red). No blocking DNA was used. 4′,6‐diamidino‐2‐phenylindole was used for counterstaining all slides. Metaphases were detected using a high‐throughput, fully automated Zeiss Axio ImagerZ2 upright epifluorescence microscope (Carl Zeiss Ltd.). Image capture was performed using a MetaSystems Coolcube 1‐m CCD camera and image analysis was carried out using Metafer4 (automated metaphase image capture) and ISIS (image processing) software (Metasystems GmbH).

## 3 RESULTS

### 3.1 Generation of chromosome‐specific SNPs

**FIGURE 1:**
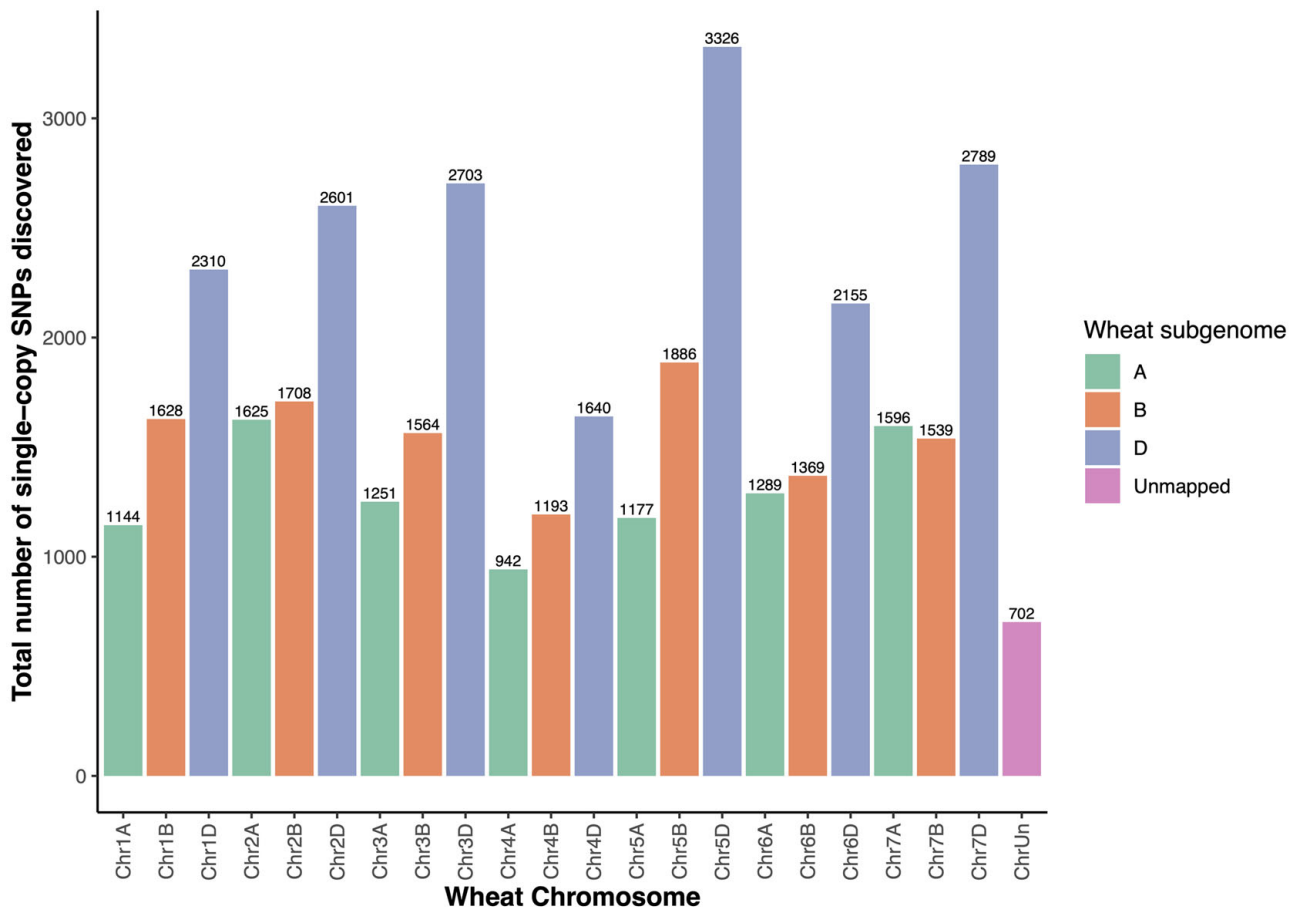
Plot showing the number of single nucleotide polymorphism (SNPs) on each wheat chromosome, identified as polymorphic between hexaploid wheat and *Am. muticum* in unique sequence regions of the wheat genome

Alignment of *Am. muticum* WGS reads against the wheat reference genome RefSeq v1.0 and filtering for good quality uniquely mapped reads led to the identification of 38,137 SNPs in unique sequence regions of the wheat genome (Supplemental Table S1). Figure 1 shows the total number of SNPs found per wheat chromosome. In each homoeologous group the D genome chromosomes were found to have the most SNPs with *Am. muticum* with Chromosome 5D having the highest number of SNPs (3,326) while chromosome 4A had the least (942).

**FIGURE 2:**
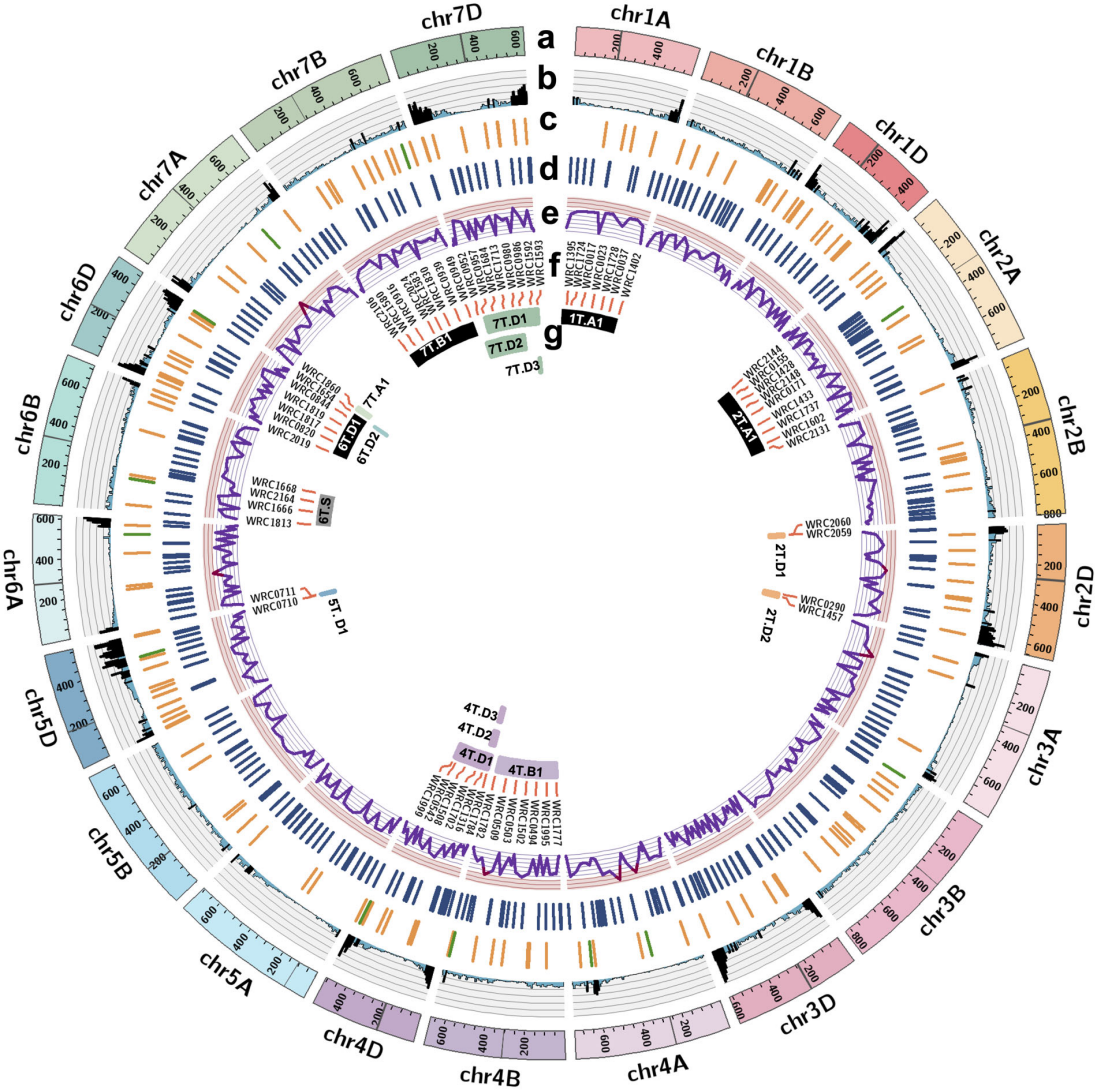
Circos plots of (a) hexaploid wheat chromosomes (200 = 200 Mbp) with horizontal lines indicating position of centromere; (b) Single nucleotide polymorphism (SNP) density in 10 Mbp bins (black = bins with >50 SNPs; starting at 0, each grid‐line on the *y* axis = 44.2 SNPs); (c) position of validated chromosome‐specific Kompetitive allele‐specific polymerase chain reaction (KASP) markers in Set 1 (orange) and Set 2 (green); (d) position of validated chromosome‐specific KASP markers in Set 3; (e) distance between adjacent KASP markers on a wheat chromosome (red line = where distance between two markers >60 Mbp; starting at 0, each grid‐line on the *y* axis = 10.31 Mbp); (f) a selection of KASP markers that detect all the introgression in the wheat–*Am. muticum* DH introgression lines; (g) introgressions in the DH lines, colored according to the corresponding recombinant wheat chromosome (black = disomic substitution, grey = disomic addition)

SNP density across 1,416 bins of 10 Mb each ranged from 0 (45 bins) to 221 SNPs (chr3D:600000000‐610000000 Mbp). Figure 2b depicts the range of SNP densities found across the wheat genome and bins with greater than 50 SNPs are shown in black. Of the 190 bins with more than 50 SNPs, 46 were present on A genome chromosomes, 19 on the B genome, and 125 on the D genome. Most of the latter were found on the distal ends of D genome chromosomes (Figure 2b).

### 3.2 Chromosome‐specific KASP marker development

Set 2 KASP markers previously developed and tested on *T. urartu* introgression lines (Grewal et al. 2021) were tested on wheat parental cultivars and *Am. Muticum* accessions in this study. Of the 224 markers in this set, 194 (∼86.6%) failed to detect the *Am. muticum* allele, 17 (∼7.6%) were monomorphic between wheat and *Am. muticum*, and 13 (∼5.8%) were found to be polymorphic between wheat and *Am. muticum* accessions used in this study. The positions on the wheat chromosomes of these 13 markers, together with the 150 from set 1, previously developed and validated to be polymorphic between wheat and *Am. muticum* (Grewal et al., 2020a), are shown in Figure 2c.

With the aim of having a KASP marker every 60 Mbp on a wheat chromosome, 698 new KASP assays were designed (Supplemental Table S2) in gap regions and tested on wheat, *Am. muticum* and three wheat–*Am. muticum* F_1_ lines in this study. Of these, 251 (∼36%) failed at the PCR stage, 49 (∼7%) did not amplify the *Am. muticum* allele, 22 (∼3.2%) were polymorphic within the wheat cultivars used as controls, and 10 (∼1.4%) were monomorphic between wheat and *Am. muticum*. Of the remaining 366 KASP markers that were polymorphic between wheat and *Am. muticum*, 31 failed to detect the *Am. muticum* allele in the heterozygous state. Thus, 335 KASP markers were validated as robust and their positions on the wheat chromosomes are indicated in Figure 2d.

In total, 498 well‐distributed, chromosome‐specific KASP markers (Supplemental Table S3), polymorphic between wheat and *Am. muticum*, were used for downstream genotyping of introgression lines. Figure 2e shows a line plot of the physical distance between these markers in wheat where each gridline of the *y* axis represents 10 Mb physical distance on a chromosome. The distance between the markers ranged from just 3 bases to ∼82.5 Mb with an average distance of 26 Mb. The average distance between the tip of the short arm and the first marker on the arm was 2.9 Mb while that from the last marker to the end of the long arm was 2.3 Mb. There were only seven instances where the gap between two KASP markers exceeded the desired 60 Mb, and these are shown with a red stroke in the line in Figure 2e. All these gaps were due to poor availability of SNPs within the desired bin as shown by the corresponding SNP density plot (Figure 2b).

### 3.3 Validation of KASP markers through genotyping of introgression lines

The set of 498 chromosome‐specific KASP markers, containing markers developed in previous studies and in this work, were used to genotype 67 DH wheat–*Am. muticum* introgression lines (King et al., 2019) along with parental wheat cultivars, *Am. muticum* accessions, and F_1_ lines as controls. Previously, these DH lines were characterized using the Axiom^®^ 36K Wheat Relative Genotyping Array and multi‐color genomic in situ hybridization (mcGISH) (King et al., 2017; King et al., 2019). The former technique provided information about what homoeologous group(s) from *Am. muticum* had introgressed into wheat and the latter identified the wheat subgenome(s) the *Am. muticum* segment(s) had recombined with and/or substituted. However, a drawback of the mcGISH technique is that it is unable to visually detect chromosome segments that are smaller than 18–20 Mbp.

In the current study, a homozygous introgression was detected through the presence of a homozygous *Am. muticum* allele called by KASP markers present in the wheat chromosome region that the segment had recombined with or substituted. The wheat allele for these markers was not called as it was replaced by the *Am. muticum* introgression in both copies of the chromosome, hence a wild/wild allele call. A homozygous introgression also produced heterozygous calls by KASP markers that were present on homoeologous chromosomal regions in wheat. These markers detect the presence of the *Am. muticum* segment, but the corresponding wheat allele in the homoeologous chromosome had not been replaced, hence a wild/wheat allele call. Through genotyping of the DH lines with these chromosome‐specific KASP markers, we were able to validate most of the previous results and, in addition, identify the specific wheat chromosomes that the introgressions from *Am. muticum* had recombined with or substituted (Table 1). The markers helped in identifying specific cases of aneuploidy in some DH lines to support the mcGISH observations but also suggested disparities with previously reported results.

**TABLE 1:** Details of the type of introgression, its code (as indicated in Figure 2g), and the wheat chromosome it had recombined with or substituted in each wheat–*Am. muticum* doubled haploid (DH) line as indicated through genotyping with chromosome‐specific Kompetitive allele‐specific polymerase chain reaction (KASP) markers. Observations about the chromosome constitution (deletions and aneuploidy) are also shown

**DH line name**	**Introgression type: Whole (W), arm (Telo), or recombinant (R)**	**Segment code** **^a^** **(A, B, D and T genomes represented as letters)**	**Wheat chromosome recombined with (if R) or substituted (if W)**	**Observations about chromosome constitution**
DH‐1	W	6T.D1	6D	
DH‐6, 7, 8, 10, 11, 13, 339	R	2T.D2	2D	
DH‐15	W, R	2T.A1, 4T.B1	2A, 4B	
DH‐16	W, R, R	2T.A1, 4T.B1, 6T.D2	2A, 4B, 6D	
DH‐17, 18, 20, 21	R, R	4T.B1, 6T.D2	4B, 6D	
DH‐19	R, R, W	4T.B1, 6T.D2, 7T.B1	4B, 6D, 7B	
DH‐28	Telo	6T.S^b^	–	
DH‐29	W	7T.B1	7D	
DH‐62, 71, 74, 348	R, R	4T.D2, 7T.A1	4D, 7A	
DH‐63, 65, 66, 76, 77, 81, 341, 360	R	4T.D2	4D	1D is missing in DH81, 7BL^b^ missing in DH‐360
DH‐83, 84, 85, 92	R	5T.D1	5D	4BL is missing in DH‐83 and DH‐92
DH‐86, 91, 94	R	2T.D1	2D	4 copies of 2D; interstitial deletion in 4A in DH‐86
DH‐89	R, R	2T.D1, 5T.D1	2D, 5D	4 copies of 2D; interstitial deletion in 4A
DH‐96, 97	R	4T.D3	4D	1DS is missing in DH‐97
DH‐121	R, R, R	4T.D1, 5T.D1, 7T.D1	4D, 5D, 7D	
DH‐122	R, R	5T.D1, 7T.D1	5D, 7D	
DH‐123	R	7T.D1	7D	
DH‐124, 126, 128, 129, 131, 134, 137–139, 141, 144, 147, 355–357	–	–	–	1AL‐1BL translocation Tetraploid for 1A Deletion of 1B not involved in 1A‐1BL translocation
DH‐161	W	1T.A1	1A	7D is missing
DH‐191, 192, 198, 202, 203	R	7T.D2	7D	
DH‐193, 195–197	R, R	7T.D2, 7T.D3	7D, 7D	

^a^ From Figure 2g.

^b^ S = short‐arm, L = long‐arm.

A subset of KASP markers that detect the *Am. muticum* introgressions present in these DH lines and the positions of these introgressions in the wheat genome are shown in Figures 2f and 2g, respectively. In total, 17 different introgressions were found to be present in these lines, using this new set of chromosome‐specific KASP markers, including four whole chromosome introgressions from 1T, 2T, 6T, and 7T, a telocentric introgression of the short arm of chromosome 6T, and 12 large and small segments from chromosomes 2T, 4T, 5T, 6T, and 7T that had recombined with various wheat chromosomes (Table 1 and Figure 2g).

### 3.4 Deviations/differences from previous characterization of DH lines

**FIGURE 3:**
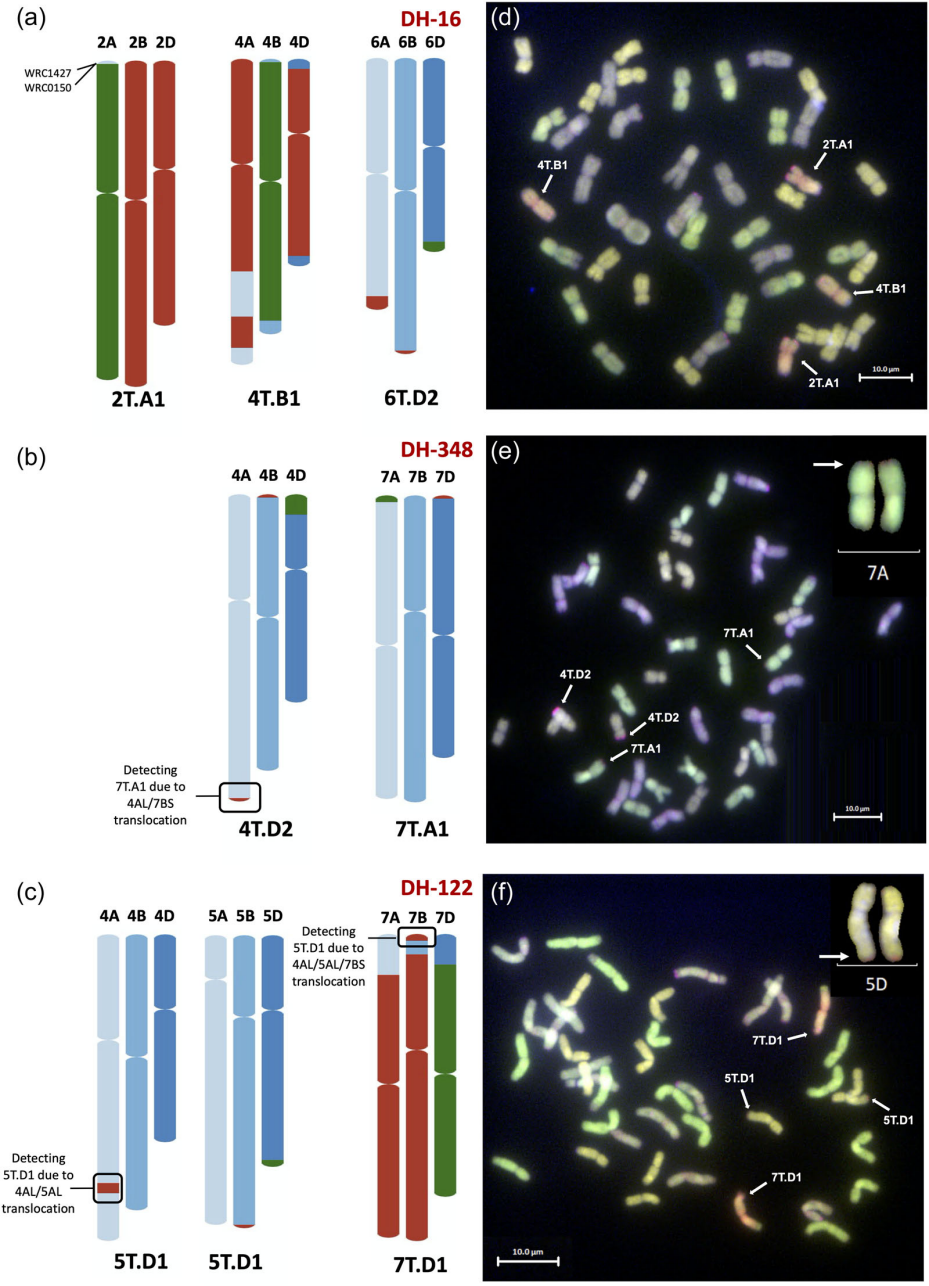
New small introgressions detected in wheat–*Am. muticum* doubled haploid (DH) introgression lines using chromosome‐specific Kompetitive allele‐specific polymerase chain reaction (KASP) markers. Graphical representation of KASP marker data detecting *Am. muticum* introgressions on wheat chromosomes in lines (a) DH‐16; (b) DH‐348; (c) DH‐122. Shades of blue represent presence of homozygous wheat alleles; red indicates heterozygous calls and green indicates presence of homozygous *Am. muticum* alleles. McGISH analysis of root metaphase spreads validating marker data in (d) DH‐16; (e) DH‐348; (f) DH‐122. Green indicates A‐genome chromosomes, blueish grey indicates B genome, yellow indicates D genome, and red indicates *Am. muticum* genome. White arrows point towards *Am. muticum* introgressed segments or chromosomes

As mentioned above, KASP markers that detect the introgression on the recombinant chromosome result in a homozygous call for the *Am. muticum* allele. In Figures 3a‐c, which show genotyping results of some of the DH lines, these homozygous *Am. muticum* calls are indicated in green. Markers on homoeologous wheat chromosomes that also detect the same introgression give heterozygous calls that are shown in red. The three wheat subgenomes are represented in shades of blue and indicate the presence of the wheat allele for KASP markers in those chromosomal regions. Figure 3b and 3c also show that heterozygous calls identifying introgressions from *Am. muticum* chromosomes were also obtained on nonhomoeologous chromosomes in wheat due to chromosome rearrangements within wheat that were not present in *Am. muticum* such as the 4/5/7 translocation (Devos et al., 1995; Dvorak et al., 2018).

Previously, DH lines 15 and 16 were characterized as having two large introgressions from *Am. muticum* chromosomes 2T and 4T, both recombined with B genome chromosomes in wheat (King et al., 2019). Genotyping of these lines in this study showed that, although chromosome 4T did recombine with chromosome 4B of wheat (4T.B1; Figure 3a), chromosome 2T was introgressed as a whole chromosome that substituted a majority of chromosome 2A of wheat (2T.A1; Figure 3a). Kompetitive allele‐specific PCR markers on the distal end of the short arm of chromosome 2A indicate that a very small segment of 2AS (∼12 Mbp) is potentially still present in these lines (Figure 3a). However, GISH indicated that the 2T was potentially introgressed as a whole chromosome due to the presence of *Am. muticum* telomeric repeat signals on both ends of this introgression (Figure 3d). If the 2AS segment had recombined with 2T or translocated onto another wheat chromosome, it would not be visible via GISH due to its small size.

The markers also showed that DH lines 16–21 had a small 6T segment (up to 10 Mb) on the distal end of 6DL (6T.D2; Figure 3a), which was not previously detected by the Axiom array and is not visible by GISH. Genotyping analysis of four other DH lines 62, 71, 74, and 348 showed that, in addition to the 4T.D2 segment, a very small segment (up to 20 Mb) from 7T was present at the distal end of 7AS (7T.A1; Figure 3b), which had not been detected before in these lines. This very small segment on the distal end of chromosome 7AS was also detected by GISH in this study (Figure 3e). The KASP markers were also able to detect another small segment (between 20–30 Mbp) from chromosome 5T in DH lines 121 and 122 (5T.D1; Figure 3c). Due to its slightly bigger size, this *Am. muticum* segment can be viewed by GISH on the distal end of chromosome 5DL in DH‐122 as shown in Figure 3f.

Genotyping analysis of 15 DH lines (codes between DH 124–147 and DH 355–357), all shown previously to have a 1T introgression on chromosome 1A (King et al., 2019), showed that no introgression from *Am. muticum* was present in these lines. The absence of any call for the majority of the KASP markers on chromosome 1B indicated that these lines had lost the pair of 1B chromosomes, but a small segment from the distal end of 1BL (∼50 Mbp) had been retained as indicated by the presence of wheat alleles for markers in this region. This new information potentially indicates that the translocation previously observed by mcGISH on a pair of 1A chromosomes and thought to be 1T was from chromosome 1BL.

## 4 DISCUSSION

Previous studies have reported chromosome‐specific KASP markers between wheat and *Am. muticum* (Grewal et al., 2020a) and other wild relative species (Grewal et al., 2020b; Grewal et al. 2021), which have been used for genotyping wheat–wild relative introgression lines. The objective of this work was to fill in the gaps with more KASP markers to increase the efficiency of genotyping by using an approach that involved faster SNP discovery and a more robust, chromosome‐specific assay design than the ones reported in previous studies. In this work, we produced ∼38K SNPs between wheat and its wild relative *Am. muticum* in unique sequence regions of the wheat genome and then converted some of these into wheat chromosome‐specific KASP markers. In combination with previously designed chromosome KASP markers, a new set of well‐distributed markers was obtained and used to re‐genotype wheat–*Am. muticum* DH introgression lines (King et al., 2019) to validate this marker set as a useful genotyping tool and detect as many *Am. muticum* introgressions as possible.

A recently developed set of KASP markers (Set 2) was tested on *Am. muticum* accessions in this study, but only 5.8% of the 224 assays were found to be polymorphic with wheat. This was as expected since this set of markers was originally developed to detect *T. urartu* introgressions in a wheat background (Grewal et al. 2021). When the 13 KASP markers were added to the 150 *Am. muticum* KASP markers developed during the original study (Grewal et al., 2020a), numerous gaps between markers were still present (Figure 2c), preventing a uniform spread of markers able to detect *Am. muticum* introgressions across the whole of the wheat genome.

### 4.1 SNP discovery

A major bottleneck at this stage was the lack of SNPs between wheat and *Am. muticum* that could be converted to KASP markers in regions that lacked an existing assay. With the advent of cheaper sequencing costs, it was possible to sequence the wild relative species to gain abundant SNPs for KASP assay design, many of which could potentially be polymorphic between wheat and other wild species. However, in polyploid crops such as bread wheat, it is challenging to generate chromosome‐specific KASP assays able to distinguish heterozygous from homozygous individuals (co‐dominant SNPs) and requires extensive validation (Allen et al., 2011; Allen et al., 2013; Grewal et al., 2020a; Makhoul et al., 2020). Thus, to avoid homoeologous SNPs, which require a cumbersome KASP assay design process involving allele ‘anchoring’ for chromosome specificity (Grewal et al., 2020a), the approach taken here was to develop a pipeline for *de novo* SNP discovery with subsequent filtering for SNPs that were in unique 100‐bp sequence regions of the wheat genome (Supplemental Figure S1). The latter step involved removal of any SNP‐containing query sequences from the final output that had a BLAST hit to a sequence(s) in the wheat genome other than itself. This resulted in ∼38K SNPs between wheat and *Am. muticum*, each assigned to a specific wheat chromosome (Figure 1). This pipeline allows for site‐specificity in the wheat genome; however, validation of this with PolyMarker resulted with hits to paralogous sequences on a wheat chromosome in rare instances.

The presence of more than 36K subgenome orphan genes has been reported in the wheat genome assembly RefSeq1.0 International Wheat Genome Sequencing Consortium (IWGSC) et al.,(2018). These were defined as subgenome‐specific genes found only in one wheat subgenome. However, this work did not target these orphan genes for SNP discovery, and thus, it was not known how many of the SNPs generated actually lay within wheat's orphan genes. In our previous work based on PCR‐amplicon based sequencing and subsequent SNP discovery (Grewal et al., 2020a), only 18.2% of the 2,374 SNP‐containing sequences were found to be in unique sequence regions of the wheat genome.

The D subgenome was found to have the most SNPs with *Am. muticum* (17,524), almost double those found with the A subgenome (9,024; Figure 1). This could possibly have been because of more unique sequence regions in the D subgenome than the A subgenome. However, the previous study suggested that the D subgenome had the least amount of orphan genes (19,523) compared with the A (22,496) and B (25,172) subgenomes International Wheat Genome Sequencing Consortium (IWGSC) et al.,(2018). Another possibility could be that *Am. muticum* is more closely related to the progenitors of the D subgenome (i.e., *Ae. tauschii;* McFadden & Sears, 1946), which resulted in more *Am. muticum* sequence reads being mapped to the D subgenome chromosomes, in turn producing more SNPs on the D subgenome. *Am. muticum* was previously classified under *Aegilops* species as *Aegilops mutica* Boiss. and like *Aegilops sharonensis* Eig could be more closely related to D genome progenitors than B genome species (Marcussen et al., 2014). Early reports of homoeology of *Am. muticum* chromosomes suggested that T genome chromosomes pair with D genome chromosomes almost regularly in F_1_ from crosses of *Am. muticum* with D genome species (Jones & Majisu, 1968), and recent reports have also shown that *Am. muticum* pairs more frequently with D and B genome chromosomes than with the A subgenome (King et al., 2017). Conversely, the increased number of SNPs with the D subgenome could be an indication of increased genetic diversity and sequence variation between the T and D genome species but not enough to prevent the sequences from being mapped onto the D subgenome.

Single nucleotide polymorphism density analysis for10 Mbp bins across the 21 chromosomes of wheat showed that SNP‐dense regions (>50 SNPs per bin) were skewed to the distal ends of the chromosomes with a majority on the D genome chromosomes as shown in black in Figure 2b. This is expected given that gene density on wheat chromosomes decreases towards centromeric regions International Wheat Genome Sequencing Consortium (IWGSC) et al.,(2018; Brinton et al., 2020; Walkowiak et al., 2020; Przewieslik‐Allen et al., 2021).

### 4.2 Development of chromosome‐specific KASP markers

A small portion of these SNPs (698) was selected to be converted into chromosome‐specific KASP markers and added to the existing set of markers to provide a diagnostic marker for *Am. muticum* every 60 Mbp on a wheat chromosome. Of these, 48% (335) were validated as robust, able to distinguish between heterozygous and homozygous introgression genotypes (Figure 2d), while ∼36% (251) failed to amplify a PCR product. In the previous study involving development of chromosome‐specific KASP markers (Grewal et al., 2020a), only 27% of the assays failed at the PCR stage. This could potentially be due to presence of sequencing errors or additional SNPs in the flanking sequence around the target SNP preventing efficient primer‐binding in those sites and/or due to sub‐optimal primer design. However, we reduced the percentage of assays that failed to detect the wild relative allele from 14.1% in the previous study to 7% in this study. The fact that *Am. muticum* is an outbreeding species and has an increased level of heterozygosity in its genome sequence could be contributing to the failure of KASP assays at the validation stage. If the target SNPs are present within the wild species and polymorphic for both the wild and wheat alleles, then it is possible that the DNA from plants used as controls for the *Am. muticum* accessions could be homozygous for the wheat allele or heterozygous at the target SNP.

We also observed 31 (∼4.4%) KASP assays that were polymorphic between wheat and *Am. muticum*, but the heterozygous F_1_ reference genotypes wrongly clustered as homozygous with the wheat parents and thus, these KASP markers were deemed unsuitable for downstream genotyping of introgression lines. A previous study looking into this false SNP call assignments for some heterozygous genotypes suggested that artificial heterozygous DNA samples from parental lines, instead of natural heterozygote plants, can be used to identify false clustering (Makhoul et al., 2020).

This new set of KASP markers filled many of the gaps that existed between the markers developed using SNPs discovered through amplicon‐ or exome‐based sequencing. The few regions where marker distances exceeded the desired 60 Mbp (Figure 2e) were due to a lack of SNPs between wheat and *Am. muticum* in those chromosomal regions. Five out of these seven regions surrounded the centromeres of chromosomes 2D, 3A, 4A, 6A, and 7A. As mentioned before, SNP density around centromeric and peri‐centromeric regions is expected to be low (Brinton et al., 2020; Walkowiak et al., 2020) due to enrichment of sequence repeats and lower sequencing depths (Choulet et al., 2014; Wen et al., 2017).

### 4.3 Genotyping of *Am. muticum* DH lines

Kompetitive allele‐specific PCR genotyping with the new marker set largely confirmed previous genotyping results of the 67 wheat–*Am. muticum* DH lines (King et al., 2019). However, there were a few cases where the genotyping analysis provided new information that either negated previous results or highlighted new introgressions not observed previously. The latter included very small introgressions that were missed in the previous study due to a lack of markers in those regions and limitations of the GISH technique. After increasing the density of KASP markers available for identifying *Am. muticum* segments in this study and using the larger marker set for genotyping these DH lines, 3 new small introgressions were found: 6T.D2 on 6DL (up to 10 Mbp), 7T.A1 on 7AS (up to 20 Mbp), and 5T.D1 on 5DL (up to 30 Mbp; Table 1, Figure 3a‐c). The chromosome‐specificity of the KASP markers allowed detection of the wheat chromosome that was involved in the recombinant chromosome or that had been substituted. Thus, it was observed that in DH lines 15 and 16, 2T.A1 was a whole chromosome that had replaced both 2A chromosomes rather than recombined with B genome chromosomes as previously reported. Where possible due to the size of the introgression, some of these results were validated by mcGISH in this work (Figure 3d‐f).

The chromosome‐specificity of these KASP markers also allowed the detection of a number of wheat chromosome deletions in the DH lines as shown in Table 1. However, these were limited to the detection of homozygous deletions. These homozygous deletions included both whole wheat chromosomes or segments (both large and small) from the wheat chromosomes. In this context, one of the main observations involved the 15 sister DH lines (codes between DH‐124 to 147 and DH‐355 to 357) that showed that the pair of 1B chromosomes had been deleted in these lines except for a small segment at the distal end of 1BL. We proposed that it was this 1BL segment that had translocated/recombined with a pair of A genome chromosomes, most likely chromosomes 1A. These lines also have 16 A genome chromosomes (King et al., 2019), so it is possible that the pair of 1A‐1BL recombinant chromosomes are present in addition to the pair of 1A chromosomes since the KASP markers at the distal end of 1A do not indicate the absence of any of the 1A wheat alleles.

## 5 CONCLUSION

The method of generating SNPs between wheat and *Am. muticum* in unique sequence regions of the wheat genome, through whole genome sequencing of the wild species, is rapid and allows for the development of robust chromosome‐specific KASP assays. A variety of wild relative species are being used to increase the genetic diversity in hexaploid wheat. This approach can therefore be applied to other wheat wild relative species for SNP discovery, highlighting the need for greater investment in whole genome sequencing of these wild species. These KASP markers have greatly increased our capability to characterize, screen, and identify both introgressions and wheat chromosomal aberrations in wheat–wild relative introgression lines. However, it is important to note that their efficiency is dependent on their density across the wheat genome and small introgressions existing between two KASP markers could have gone undetected. With the reducing cost of DNA sequencing, we envisage that the next improvement in characterization of such introgressions, with the potential to give higher resolution, would be low‐coverage whole genome resequencing of wheat–wild relative introgression lines as has been recently demonstrated (Coombes et al., 2021).

## AUTHOR CONTRIBUTIONS

Surbhi Grewal: Conceptualization; Data curation; Formal analysis; Investigation; Methodology; Project administration; Validation; Writing‐original draft; Writing‐review & editing. Benedict Coombes: Data curation; Formal analysis; Investigation; Methodology. Ryan Joynson: Formal analysis; Investigation; Methodology. Anthony Hall: Methodology; Resources; Supervision. John Fellers: Resources; Writing‐review & editing. Duncan Scholefield: Data curation; Investigation. Stephen Ashling: Data curation; Investigation. Peter Isaac: Methodology. Julie King: Conceptualization; Funding acquisition; Project administration; Resources; Supervision; Writing‐review & editing.

## CONFLICTS OF INTEREST

Peter Isaac was employed by the company iDna Genetics Ltd. Ryan Joynson was employed by Earlham Institute while this work was carried out. The remaining authors declare that the research was conducted in the absence of any commercial or financial relationships that could be construed as a potential conflict of interest.

## Supporting information


**Supplemental Figure S1**. A diagrammatic representation of the *de novo* pipeline for SNP discovery starting with whole genome sequence reads of *Amblyopyrum muticum* that are mapped to wheat cultivar Chinese Spring RefSeq1.0 assembly (International Wheat Genome Sequencing Consortium (IWGSC) et al., 2018).

**Supplemental Table S1** Detailed list of all 38,137 chromosome‐specific SNPs discovered between bread wheat and *Amblyopyrum muticum*
**Supplemental Table S2** Detailed list of 698 KASP assays developed in this study for validation in wheat
**Supplemental Table S3** Detailed list of 498 validated chromosome‐specific KASP assays diagnostic for *Am. muticum* in wheat and their physical position in wheat (RefSeq v1.0)

## Data Availability

Raw reads data for *Am. muticum* have been made available through the Sequence Read Archive via Project PRJNA783544 and through the Grassroots data repository hosted by the Earlham Institute and funded by DFW programe (https://opendata.earlham.ac.uk/wheat/under_license/toronto/Grewal_et_al_2021‐09‐13_Amybylopyrum_muticum/). The latter also hosts the raw and filtered vcf files generated in this study. The code and custom scripts for the entire pipeline is available via https://github.com/Surbhigrewal/chromosome‐specific_KASPs. All DH lines used in this study are available through the Germplasm Resource Unit at the John Innes Centre. The genotyping data are available from the corresponding author on reasonable request.
