# Supplementary material for: Chromosome‐specific KASP markers for detecting Amblyopyrum muticum segments in wheat introgression lines
Source: Plant Genome. 2022 Jan 31;15(1):e20193. doi: 10.1002/tpg2.20193 (PMC12807185; doi:10.1002/tpg2.20193)
Supplement: Supplementary file 1 [file TPG2-15-e20193-s002.docx]

**Figure S1**. A diagrammatic representation of the *de novo* pipeline for SNP discovery starting with whole genome sequence reads of *Amblyopyrum muticum* which are mapped to wheat cv. Chinese Spring RefSeq1.0 assembly (Appels et al. 2018). This is followed by filtering steps using samtools that remove duplicates, unmapped and non-uniquely mapped reads. Variants are called with bcftools and further filtration is performed using custom scripts that leads to chromosome-specific SNPs suitable for KASP assay design. Created by biorender.com
